# Environmental estrogens and animal reproductive health: mechanisms, biomarkers, and intervention approaches

**DOI:** 10.1186/s40104-026-01427-6

**Published:** 2026-06-01

**Authors:** Shuang Liang, Jianfeng Ma, Siyu Chen, Xia Dong, Jiawei Lu, Yan Zhu, Lei Chen, Yan Wang, Lili Niu, Ye Zhao, Linyuan Shen, Li Zhu, Mailin Gan

**Affiliations:** 1 Farm Animal Germplasm Resources and Biotech Breeding Key Laboratory of Sichuan Province, Sichuan Agricultural University, Chengdu, 611130 China; 2 State Key Laboratory of Swine and Poultry Breeding Industry, Sichuan Agricultural University, Chengdu, 611130 China; 3 Key Laboratory of Livestock and Poultry Multi-Omics, Ministry of Agriculture and Rural Affairs, College of Animal and Technology, Sichuan Agricultural University, Chengdu, 611130 China

**Keywords:** Animal reproduction, Endocrine disruption, Environmental estrogens, Reproductive toxicity

## Abstract

Environmental estrogens, a group of exogenous endocrine-active compounds, are frequently present in animal feed, water, and husbandry environments. These compounds, which include synthetic xenoestrogens, phytoestrogens, and metalloestrogens, can disrupt reproductive physiology and developmental processes in domestic animals. This review examines their effects on key reproductive outcomes such as folliculogenesis, spermatogenesis, gamete quality, and overall fertility. We discuss the underlying molecular mechanisms, focusing on hormone receptor signaling and epigenetic modifications, and highlight the particular relevance of low-dose and developmental stage-specific exposure in breeding populations. Additionally, potential mitigation approaches are outlined, including nutritional strategies, feed management, and genetic or biotechnological interventions aimed at preserving reproductive performance and animal health. This review elucidates the reproductive toxicological mechanisms of environmental estrogens and provides a scientific basis for developing effective prevention and mitigation strategies.

## Introduction

The acceleration of industrialization and modernization has led to the widespread application of numerous synthetic organic chemicals in agriculture, industrial production, and daily life. While these substances offer significant convenience, they also pose serious threats to the ecological environment and human health. Environmental estrogens (EEs), a prominent class of endocrine-disrupting chemicals (EDCs) [1, 2], have garnered increasing attention in environmental medicine, toxicology, and public health due to their ability to mimic, antagonize, or otherwise interfere with the physiological functions of endogenous estrogens [3, 4]. These compounds are commonly found in industrial wastewater, pesticide residues, plastics, personal care products, detergents, and food packaging materials. Representative EEs include bisphenol A (BPA), phthalates, polychlorinated biphenyls (PCBs), polybrominated diphenyl ethers (PBDEs), nonylphenol, and the synthetic estrogen diethylstilbestrol (DES).

Environmental estrogens are a group of environmentally pervasive chemicals that can disrupt the endocrine system by mimicking or blocking the action of natural estrogens. They bind to intracellular estrogen receptors (ERα and ERβ), activating or inhibiting downstream signaling pathways and thereby disturbing the homeostasis of the hormonal regulatory network [5, 6]. Beyond these classical nuclear receptor-mediated pathways, EEs can also activate non-genomic signaling via membrane receptors, notably GPER. This triggers pathways such as MAPK/ERK and PI3K/Akt, which rapidly modulate cell metabolism, proliferation, and differentiation [7, 8]. Furthermore, EEs can interact with other nuclear receptors—including the androgen receptor (AR), progesterone receptor (PR), and thyroid hormone receptor (TR)—potentially disrupting multiple hormonal pathways and compromising the dynamic balance of the endocrine system [6, 9]. At the molecular level, EEs can interfere with transcription factor activity, alter chromatin structure, and induce oxidative stress and inflammatory responses, thereby affecting cellular function and gene expression [10–12]. Recent studies also indicate that EEs can modulate the diversity and composition of the gut microbiota, indirectly interfering with host metabolic homeostasis and immune regulation. This emerging pathway offers a novel mechanistic perspective on their systemic toxicity [13, 14]. Given the crucial role of estrogen in numerous physiological processes, exposure to EEs may disrupt individual endocrine homeostasis and contribute to a variety of health disorders. Notably, these substances exhibit two key characteristics: the "low-dose effect", where significant biological responses can occur at very low concentrations, and the "critical window of exposure effect", meaning that exposure during sensitive developmental stages—such as the embryonic, infant, and pubertal periods—is more likely to cause persistent or even irreversible physiological damage [15].

Among the various physiological systems susceptible to EEs, the reproductive system is considered one of the primary targets due to its high sensitivity. Extensive evidence indicates that EEs can disrupt normal reproductive development and function through multiple mechanisms, exerting adverse effects on both male and female reproductive systems at various levels. In males, EEs are associated with testicular developmental abnormalities, reduced sperm count and quality, impaired testosterone synthesis, and dysregulation of androgen levels, ultimately compromising fertility [16–18]. In females, exposure has been linked to ovarian dysfunction, ovulation disorders, abnormal endometrial responses, and menstrual cycle irregularities, potentially resulting in infertility in severe cases [4, 19, 20]. Moreover, exposure to EEs has been closely associated with an increased incidence of hormone-dependent cancers, such as breast, endometrial, and testicular cancer [21–23]. A growing body of research suggests that these compounds can influence the expression of reproductive genes via epigenetic mechanisms—including DNA methylation and non-coding RNA regulation—thereby potentially inducing transgenerational toxic effects [24, 25].

It is important to note that the toxicity of EEs is not confined to the reproductive system. They can also exert broad effects on other vital physiological systems. Within the endocrine system, EEs can interfere with the secretion and function of thyroid hormones, insulin, and adrenal corticosteroids, contributing to metabolic disorders, obesity, and type 2 diabetes [26, 27]. Regarding the nervous system, some EEs can cross the placental barrier, disrupt fetal neurodevelopment, and have been associated with an increased risk of childhood disorders such as attention deficit hyperactivity disorder and autism spectrum disorder [28–30]. In the immune system, these chemicals may suppress immune function and cause dysregulation, elevating susceptibility to allergies, autoimmune diseases, and infections [31]. In the cardiovascular system, EEs have been implicated in the disruption of lipid metabolism and vascular endothelial function, which are associated with the development of hypertension and atherosclerosis [32].

In summary, environmental estrogens exert extensive and profound impacts on human health through diverse mechanisms. Their effects on the reproductive system are particularly consequential, manifesting not only as diminished individual fertility but also potentially causing multi-generational reproductive decline via epigenetic mechanisms. This poses a significant threat to population stability and sustainability, underscoring the public health importance of this issue. Therefore, this review aims to systematically outline the major categories, sources, and exposure routes of EEs; elucidate the core mechanisms by which they disrupt the endocrine signaling network, including both receptor-mediated and non-receptor-dependent pathways; comprehensively describe their toxic effects on male and female reproductive systems and the underlying molecular basis; and discuss potential intervention strategies and challenges in risk assessment.

## An overview of environmental estrogens

### Mechanisms of action and major categories

Environmental estrogens, also known as xenoestrogens, constitute a class of natural or synthetic exogenous chemicals capable of mimicking, blocking, or otherwise interfering with the biological activity of endogenous estrogens. They disrupt endocrine homeostasis by activating intracellular nuclear estrogen receptors (ERs) and the membrane-associated G protein-coupled estrogen receptor (GPER), while simultaneously interfering with hormone metabolic processes and epigenetic regulation. This multifaceted disruption ultimately leads to abnormal development and functional disorders of the reproductive system. Research demonstrates that EEs are widely distributed across various environmental media and in daily consumer products such as plastics, medical devices, and personal care items, entering the body through multiple pathways including the digestive tract, respiratory tract, and skin [33–35]. Their generally hydrophobic and lipophilic nature predisposes them to accumulate in lipid-rich tissues like the breast, skin, adipose tissue, liver, and brain [36]. Beyond inducing reproductive system abnormalities and dysfunction, EEs can also interfere with central nervous system development, immune regulation, and metabolic homeostasis, exhibiting multi-system and multi-organ toxic effects [37]. Given their ubiquitous presence and potential health risks, environmental estrogens have been classified as priority endocrine disrupting chemicals (EDCs) for control by the World Health Organization (WHO) and regulatory agencies in numerous countries.

**Fig. 1:**
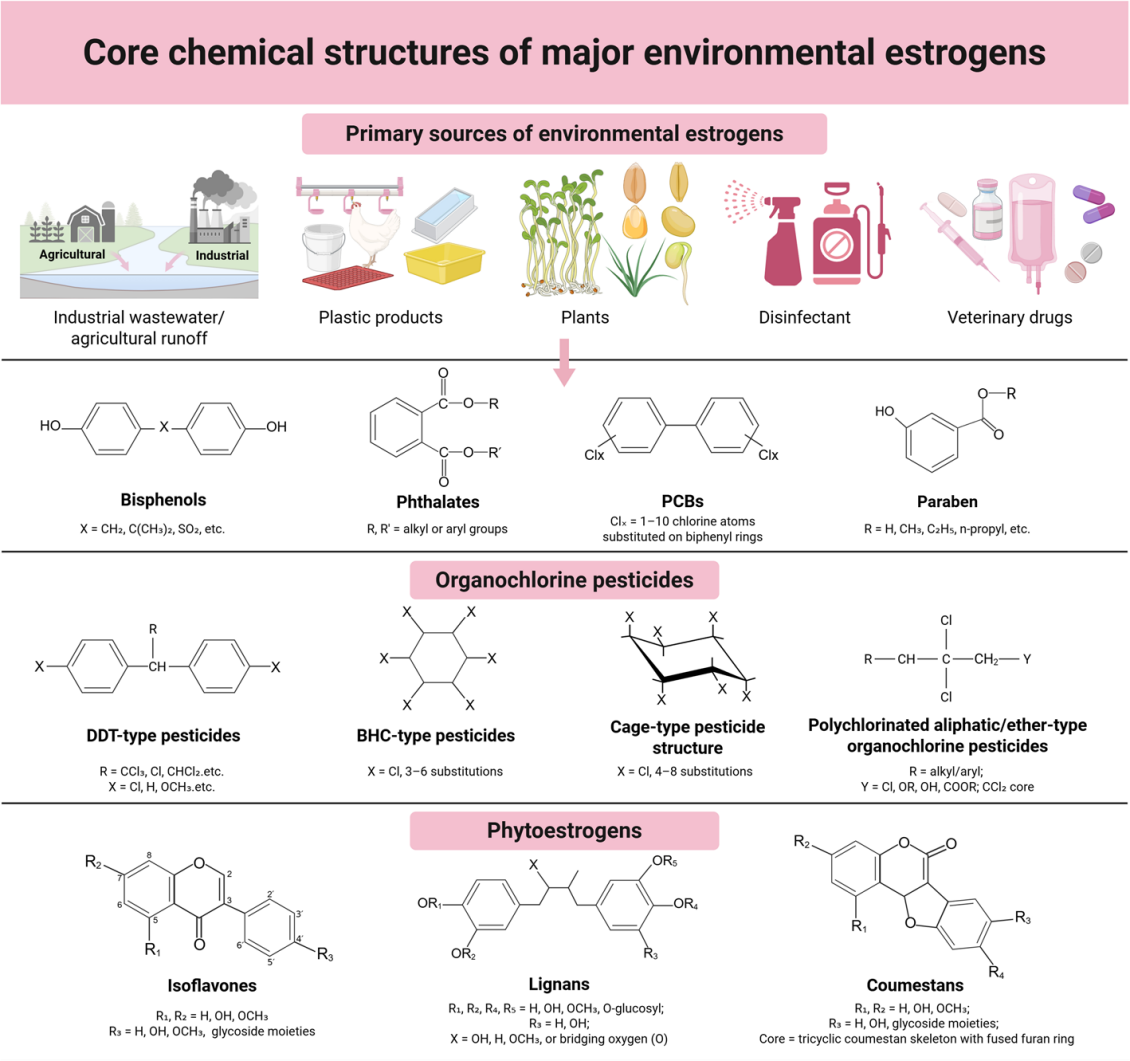
Core chemical structures of major environmental estrogens

The core chemical structures of representative environmental estrogens from each major category are illustrated in Fig. 1. Based on origin and chemical properties, environmental estrogens are primarily categorized into three groups: synthetic xenoestrogens, phytoestrogens, and metalloestrogens. Among these, synthetic xenoestrogens (e.g., bisphenols, phthalates, PCBs, organochlorine pesticides, and parabens) are ubiquitous in modern industrial and agricultural production and consumer goods, representing the most extensively studied category.

Within synthetic xenoestrogens, bisphenol compounds have attracted significant attention due to their widespread use and high potential for human exposure. Recent biomonitoring studies report high detection rates for these substances in populations globally [38–40]. As important industrial raw materials, they are extensively used in producing polycarbonate plastics, epoxy resins, thermal paper, and food-contact materials. The phenolic hydroxyl groups in their molecular structure enable them to mimic the endogenous estrogen 17β-estradiol by binding to estrogen receptors (ERα and ERβ), thereby disrupting normal endocrine regulation. This interaction confers significant estrogenic activity alongside some anti-androgenic effects. Studies indicate that their representative compound, BPA, may provoke various reproductive toxic responses. In females, BPA is associated with endometrial hyperplasia and impaired follicular development; in males, it has been linked to reduced spermatogenesis and structural changes in testicular tissue [41–43]. As understanding of BPA's health risks has deepened, bisphenol S (BPS) and bisphenol F (BPF) have been rapidly developed as substitutes and widely incorporated into products. However, both animal and cellular studies reveal that these alternative compounds exhibit endocrine-disrupting effects on hormonal regulation and gonadal development similar to BPA [44, 45]. Furthermore, recent research suggests that BPS and BPF may be metabolized less efficiently than BPA, leading to greater accumulation in adipose tissue, prolonged biological activity, and potentially increased reproductive toxicity risks [46, 47].

Phthalates (e.g., DEHP, DBP), serving as crucial plasticizers, are widely used in polyvinyl chloride (PVC) plastics, medical devices, toys, and food packaging. The ester groups in their structure confer high lipophilicity, facilitating their release from products into the environment and subsequent entry into the human body via skin contact, ingestion, or inhalation. Research shows that phthalate metabolites can inhibit key enzymes (e.g., 5α-reductase, CYP17) in the steroid synthesis pathway, thereby disrupting normal testosterone synthesis. This disruption is associated with reproductive toxicity in males, including testicular developmental abnormalities, impaired spermatogenesis, and reduced gamete quality [48–50]. Animal evidence also indicates that maternal exposure during pregnancy to these substances may cause disordered sexual differentiation and genital developmental defects in fetuses, reflecting their significant developmental toxicity and endocrine-disrupting properties during embryogenesis [51, 52].

Polychlorinated biphenyls and organochlorine pesticides (e.g., DDT and its metabolite DDE) are typical persistent organic pollutants. Their high chemical stability and strong bioaccumulation potential have led to widespread detection in marine mammals, polar birds, and human adipose tissue. Studies indicate that PCBs can disrupt endocrine homeostasis by activating the aryl hydrocarbon receptor (AhR). This activation interferes with AhR-ER signaling crosstalk and contributes to reproductive and developmental disorders [53]. Specifically, AhR activation can negatively regulate ER-mediated transcriptional activity, suppressing normal estrogen signal transduction and affecting critical physiological processes like oocyte maturation, which forms a key molecular basis for PCB reproductive toxicity [54]. In contrast, organochlorine pesticides, as another class of stable and widely distributed persistent organic pollutants, primarily exert reproductive toxicity in aquatic ecosystems. Research shows they can induce abnormal vitellogenin expression in male fish and cause testicular structural alterations and reduced reproductive capacity, suggesting potential systemic and long-term impacts on aquatic environments [55, 56]. Beyond persistent organic pollutants, short-lived metabolic endocrine disruptors like parabens have gained prominence in environmental health research due to their extensive use in consumer products and weak estrogen-like activity. As common preservatives, they are primarily used in cosmetics, skincare products, personal care items, and pharmaceuticals. Studies demonstrate that parabens can activate the ER signaling pathway, thereby affecting normal hormonal response processes [57, 58]. In mouse models, high-dose paraben exposure induces hyperproliferation of mammary epithelial cells and disrupts the secretion of endogenous hormones such as estrogen or gonadotropins [59]. Some epidemiological studies have observed correlations between internal paraben levels and abnormal expression of estrogen-sensitive genes in breast tissue, although causal mechanisms remain unelucidated [60]. At most environmentally relevant exposure doses (ng/g level), the individual effects of parabens are relatively weak; their potential health risks likely depend on synergistic interactions with other environmental endocrine disruptors (e.g., BPA) or long-term cumulative effects [61].

Phytoestrogens are a class of natural non-steroidal compounds widely distributed in various plants, primarily including isoflavones, lignans, and coumestans. Their molecular structures typically contain aromatic rings and phenolic hydroxyl groups, conferring conformational similarity to endogenous estrogens. Consequently, these compounds can bind to estrogen receptor beta (ERβ) and exert either agonistic or antagonistic effects depending on dosage and physiological context, reflecting typical bidirectional regulatory properties. Substantial clinical and animal research indicates that moderate intake of phytoestrogens can help alleviate perimenopausal symptoms, increase bone mineral density, and improve lipid and glucose metabolism [62–64]. However, exposure to high doses during critical developmental windows such as the fetal period or puberty may interfere with the normal development of the hypothalamic-pituitary-gonadal (HPG) axis, leading to abnormal gonadal differentiation and disrupted sex hormone synthesis [65, 66]. Furthermore, studies have found that some ruminants grazing on phytoestrogen-rich pasture exhibit estrous cycle irregularities and decreased fertility [67–69]. This suggests that even naturally derived phytoestrogens may pose a potential threat to animal reproductive health and ecosystem stability under specific exposure conditions.

Metalloestrogens represent an environmentally relevant estrogen subtype that has garnered increasing attention, with typical examples including heavy metal ions like cadmium (Cd), aluminum (Al), and arsenic (As). Although lacking the classic steroidal structure, certain metal ions can significantly disrupt estrogen homeostasis through multiple mechanisms. Research finds these ions can bind with high affinity to the ligand-binding sites of ERα and ERβ, activating transcription of downstream genes mediated by estrogen response elements (EREs). Simultaneously, they can regulate the gene expression and enzymatic activity of key steroidogenic enzymes such as aromatase and 17β-hydroxysteroid dehydrogenase (17β-HSD), disrupting the dynamics of endogenous estrogen synthesis and metabolism [70–73]. Additionally, these metalloestrogens can induce excessive intracellular reactive oxygen species (ROS) production, triggering oxidative stress-related damage including DNA damage and chromatin abnormalities, ultimately interfering with the stable expression and regulation of estrogen-dependent genes [74]. Abundant animal experimental evidence indicates that molecular abnormalities mediated by metalloestrogens can significantly impair reproductive system development and function. Toxic effects may manifest as testicular atrophy, decreased sperm count and motility in males, and impaired follicular development, luteal insufficiency, and hormonal cycle disruptions in females [75, 76]. Concurrently, studies note that long-term, low-dose exposure to metals like cadmium, aluminum, or arsenic may be positively associated with the risk of developing estrogen-dependent cancers such as breast and endometrial cancer [77].

### Reproductive health hazards

Despite their structural diversity and varied sources, EEs share a common potential to disrupt reproductive health. Emerging evidence suggests that EEs may induce comparable adverse outcomes in both human populations and animal models. The reproductive toxicity of major EEs across sexes and their primary molecular targets are summarized in Table 1.

**Table 1:** Reproductive toxicity and molecular targets of common environmental estrogens in males and females

Environmental estrogen	Male reproductive hazards	Female reproductive hazards	Molecular targets	Reference
Bisphenol A (BPA)	Blood-testis barrier disruption; spermatogenic cell apoptosis	Atypical hyperplasia of mammary duct epithelium; Meiotic spindle abnormalities in oocytes	ERβ-specific agonism; Aberrant H19 gene methylation	[4, 78, 79]
Phthalates (DEHP)	Vacuolar degeneration of Leydig cells; Sperm morphological abnormalities and reduced motility	Ovarian weight reduction; Accelerated primordial follicle depletion	PPARγ-mediated suppression of testosterone synthesis	[80–82]
Polychlorinated biphenyls (PCBs)	Reduced sperm density (< 15 × 10^6^/mL, WHO 2023 threshold); Increased incidence of testicular cancer	Elevated endometriosis risk; Earlier menarche onset	AhR/ARNT pathway activation; Indirect disruption of androgen signaling via estrogenic or anti-androgenic effects	[83–85]
Organochlorine pesticide metabolite (DDT/DDE)	Significant reduction in sperm concentration; Seminiferous tubule atrophy with spermatogenic cell apoptosis; Decreased serum testosterone levels	Elevated breast cancer risk; Accelerated follicular depletion	p,p'-DDE: Competitive androgen receptor (AR) antagonist; o,p'-DDT: Estrogen receptor (ER) agonist	[83, 86, 87]
Soy Isoflavones	High-dose exposure reduces testicular weight in animal models; Modulates spermatogenesis via ERβ-mediated pathways	High-dose exposure may impair ovarian follicle development and reduce serum estradiol	ERβ-selective binding affinity (Kd: 0.05–0.1 nmol/L for ERβ vs. 0.1–0.5 nmol/L for ERα)	[88–90]
Zearalenone (ZEA)	Decreased serum testosterone; Seminiferous tubule degeneration	Hyperestrogenism (e.g., vulvar swelling) in swine; Impairs oocyte quality and follicular development	Partial agonist for ERα (relative affinity: 1%–10% of E2) and ERβ; ERβ partial agonism; ROS-mediated apoptosis	[91–93]
Cadmium (Cd)	Blood-testis barrier disruption; Acute exposure induces testicular necrosis and hemorrhage; Inhibits Leydig cell steroidogenesis via 3β-HSD suppression	/	Indirect estrogenic effects via membrane receptors; Predominantly heavy metal toxicity(ROS, mitochondrial dysfunction); Epigenetic dysregulation (e.g., m^6^A methylation imbalance)	[94, 95]
Lead (Pb)	Elevated blood lead levels (e.g., > 40 μg/dL) are associated with decreased sperm count and impaired semen parameters (e.g., reduced concentration, motility, and morphology); Occupational lead exposure at high levels increases sperm DNA fragmentation via oxidative stress	/	Inhibition of steroid synthesis via oxidative stress (e.g., reduction in 3β-HSD activity); Lead-induced mitochondrial apoptosis via oxidative stress (ROS) involves downregulation of Bcl-2 and P53, overexpression of TNFα, and activation of Caspase-3, leading to DNA breaks	[96, 97]
Diethylstilbestrol (DES)	Possible increased testicular cancer risk; Increased cryptorchidism	Substantially increased vaginal clear-cell adenocarcinoma risk; Tubal abnormalities leading to ectopic pregnancy	Sustained ERα activation; Dysregulation of HOXA10 expression	[98, 99]

Substantial epidemiological evidence has linked chronic exposure to environmental endocrine disruptors to a range of adverse reproductive outcomes in humans, including impaired spermatogenesis in males, diminished ovarian reserve in females, and an increased risk of infertility [100, 101]. However, these observational associations warrant cautious interpretation due to inherent methodological limitations, such as potential confounding factors, challenges in accurately assessing long-term exposure, and heterogeneity in study populations and outcome measures. Although consistency across multiple cohorts strengthens the plausibility of a causal relationship, definitive causal inference in human studies remains constrained by these issues. Therefore, incorporating mechanistic evidence from controlled experimental models is essential to substantiate the epidemiological associations and elucidate the underlying biological pathways.

To elucidate these mechanisms, research at molecular, cellular, and organismal levels has been employed. At the cellular level, EEs can disrupt key signaling pathways and physiological functions within reproductive tissues. For example, BPA and its common substitute BPS have been shown to impair ovarian somatic cell function, as evidenced by disrupted activity in porcine granulosa cells [102]. Similarly, perfluorooctanoic acid (PFOA) induces oxidative stress and compromises steroidogenesis in these cells [103]. During early development, EEs such as BPA and its analogs may target primordial germ cells (PGCs), perturbing their migration and altering population size in zebrafish embryos; such dysregulation of chemokine signaling has been proposed to contribute to subsequent gonadal defects [104, 105]. At the gamete level, BPA exposure is associated with disrupted oocyte-cumulus cell communication and impairs meiotic progression [106]. In males, it is associated with increased sperm DNA fragmentation and morphological abnormalities, collectively compromising fertilization potential [107, 108]. Collectively, these in vitro findings provide important mechanistic insights into the cellular targets of EEs. However, it is crucial to acknowledge that while cell-based models offer a high degree of experimental control, they cannot fully replicate the complex endocrine interactions and metabolic processes of an intact organism. The reproducibility of these observations across diverse cell types and species (e.g., porcine, zebrafish, rodent) strengthens the argument for their biological relevance. Nevertheless, careful consideration is needed when extrapolating in vitro effective concentrations to realistic in vivo exposure scenarios, as factors such as bioavailability, metabolism, and tissue distribution critically determine the actual dose reaching target organs.

Collectively, these cellular disruptions can manifest as reproductive pathologies in whole-animal models. In mammals, neonatal exposure to BPS induces testicular damage comparable to that caused by BPA [109]. Phthalates, such as DBP and DEHP, are established inducers of developmental defects, including testicular injury [110, 111]. Further evidence from livestock models indicates that exposure to bisphenol AF (BPAF) can impair uterine receptivity and compromise embryo implantation; mechanistic studies in dairy goats suggest that this effect may be attributable to BPAF-induced cytotoxicity and oxidative stress in endometrial epithelial cells [112]. In amphibians, exposure to bisphenol B disrupts testis differentiation partly via the estrogen receptor-mediated pathway and subsequently causes testicular dysgenesis in *Xenopus laevis* [113]. The consistent observation of reproductive pathologies across diverse mammalian and non-mammalian species provides compelling evidence that EEs pose a genuine hazard to reproductive health. This evidence is further corroborated by the robust manifestation of testicular and ovarian defects following exposure at various life stages (e.g., neonatal, juvenile, adult) and via different administration routes. Nonetheless, direct comparisons between studies are hampered by significant heterogeneity in experimental design, particularly in dosing regimens, exposure durations, and endpoints assessed. Moving forward, the standardization of key experimental protocols would substantially enhance the robustness of the evidence base, facilitating more reliable cross-species extrapolation and human health risk assessment.

Indeed, as the preceding evidence demonstrates, the reproductive toxicity of EEs is remarkably conserved across vertebrate species. In fish, exposure to BPA, PFOA, or parabens disrupts endocrine function, reduces fertility, and can induce gonadal intersex and aberrant vitellogenin expression in males [114, 115]. In birds, the DDT metabolite p,p'-DDE causes eggshell thinning by interfering with calcium metabolism, a well-established mechanism contributing to reproductive failure and population decline [116]. The consistent observation of phenotypes such as reduced gamete quality, gonadal malformations, and decreased hatchability across diverse taxa provides a robust experimental basis for hazard assessment. One of the most robust and widely documented effects is the induction of vitellogenin in male fish, a thoroughly validated and reproducible biomarker of estrogenic exposure. Its sensitivity has been confirmed in hundreds of studies across numerous fish species, establishing it as a cornerstone of ecotoxicological screening. Building on this foundation, Table 2 provides a comparative summary of in vitro and in vivo evidence for major EEs, integrating data on estrogenic potency, environmental exposure levels, and key reproductive outcomes. By comparison, evidence for transgenerational reproductive effects, while compelling, is currently derived from a more limited set of model organisms. Further research, particularly longer-term multigenerational exposures under environmentally relevant conditions, is necessary to confirm the persistence and full manifestation of these effects. A critical next step, therefore, is to evaluate whether the effective doses observed in such controlled studies are relevant to real-world exposure scenarios.

**Table 2:** Comparison of in vitro and in vivo estrogenic effects, environmental exposure levels, and reproductive outcomes of representative environmental estrogens

Environmental estrogen	In vitro findings	In vivo effects	Exposure level context	Reference
Bisphenol A (BPA)	ERα activation (zfERα reporter): EC_50_ = 469 nmol/L; 79% efficacy vs. E2	Mammary gland development (CD-1 mice, gestational): Altered ductal architecture in female offspring at 25–250 µg/kg/d	Human: ng/mL in urine/serum Experimental dose: Higher than typical exposure, yet shows low-dose effects	[117–119]
4-Nonylphenol (4-NP)	ER agonist (Rainbow trout hepatocytes): Exhibits estrogenic activity but is approximately 2,000 to 3,000 times less potent than 17β-estradiol (E₂) at stimulating vitellogenin production	Vitellogenin induction & testicular growth inhibition (Rainbow trout): Exposure to concentrations as low as 20–50 µg/L induces vitellogenin synthesis in males and can inhibit testicular growth	Acute toxicity (fish): Reported 96-h LC₅₀ values for fish vary widely, typically in the range of 17–3,000 µg/L Environment: Detected in wastewater, with concentrations often in the µg/L range (e.g., influent levels can be elevated)	[120]
17α-Ethinylestradiol (EE2)	In transgenic zebrafish (cyp19a1b-GFP) embryos, EE2 induces brain aromatase expression with an EC₅₀ ranging from 0.01 to 0.1 nmol/L, demonstrating higher relative potency than 17β-estradiol (E₂)	Chronic exposure to environmentally relevant concentrations (e.g., 5 ng/L) can lead to complete reproductive failure and population collapse in fish. In adult zebrafish, EE2 exposure (≥ 3 ng/L) strongly induces vitellogenin synthesis and causes testicular histopathology	Typically detected in surface waters at ng/L (ppt) levels. The chronic no-observed-effect concentration (NOEC) for population-relevant endpoints in fish is below 1 ng/L, while effective concentrations for vitellogenin induction can be as low as 1–3 ng/L	[121, 122]
Genistein (Gen)	In transgenic zebrafish (cyp19a1b-GFP) embryos, its estrogenic activity (induction of GFP/brain aromatase) has an EC₅₀ of approximately 3,545 nmol/L (cyp19a1b mRNA) and 2,166 nmol/L (image analysis), confirming much lower potency than natural or synthetic estrogens like E2 and EE2	Exhibits biphasic effects: beneficial at moderate dietary levels but can cause developmental toxicity (e.g., pericardial edema, yolk sac edema, spinal kyphosis) in zebrafish embryos at high concentrations (e.g., 0.25 × 10⁻^4^ mol/L). In-utero exposure is associated with adverse effects on offspring health in animal models. Shows antagonistic effects when co-exposed with 17β-estradiol (E_2_) on cyp19a1b induction in transgenic zebrafish models	Human plasma concentrations can reach low micromolar levels (e.g., peak plasma levels of ~4 µmol/L) following consumption of soy-rich meals. Environmental concentrations in surface waters are generally low (ng/L to low µg/L range), as it is primarily of dietary origin	[123–125]
Di(2-ethylhexyl) phthalate (DEHP)	In the in vitro ER-CALUX reporter gene assay, DEHP shows very weak direct ER agonist activity, with an EC₅₀ > 30,000 nmol/L	Gestational exposure in rats (750 mg/kg/d) disrupts male reproductive tract differentiation by suppressing fetal testosterone synthesis, leading to anti-androgenic effects such as reduced anogenital distance and testis weight	Ubiquitous in plastics; its metabolites (e.g., MEHP, MEHHP, MEOHP) are commonly detected in human urine. The in vivo toxic dose (750 mg/kg/d) is much higher than typical human exposure (e.g., 95th percentile intake 21–25 µg/kg/d)	[126–128]
Polychlorinated biphenyl congener 153 (PCB-153)	In recombinant yeast estrogen screens (YES), certain PCB congeners (e.g., 2,4,6-trichlorobiphenyl) exhibit weak ER agonist activity. However, the di-ortho-substituted hexa-chlorobiphenyl PCB-153 shows negligible direct estrogenic activity in such assays. Instead, several studies report it lacks detectable agonism and can act as an antagonist, inhibiting 17β-estradiol-induced responses in vitro	As a classic persistent organic pollutant (POP), most reproductive toxicity data are derived from mixture studies rather than from the pure congener. In the E-Screen bioassay, PCB-153 (along with other highly chlorinated congeners like PCB-138 and 180) does not induce estrogenic responses but can weakly suppress estradiol-stimulated cell proliferation, indicating anti-estrogenic potential. Other investigations confirm that highly bioaccumulative PCB congeners can produce anti-estrogenic effects via receptor-mediated mechanisms	Owing to historic production and extreme environmental persistence, PCB-153 is widely detected in environmental matrices and biota, including humans. It is consistently reported as one of the most abundant PCB congeners in human adipose tissue and serum, often at concentrations in the ng/g lipid range	[129–131]
Cadmium (Cd)	Cd^2+^ ions can bind to and activate estrogen receptor alpha (ERα) in a manner distinct from the natural ligand 17β-estradiol, likely by inducing conformational changes in the receptor's ligand-binding domain	Exposure to cadmium in experimental animals (e.g., rodents) induces testicular toxicity, characterized by reduced testicular weight, decreased sperm count and motility, and histopathological damage. The reproductive toxicity is strongly associated with the induction of oxidative stress and apoptosis in testicular tissue	Human exposure occurs primarily through contaminated food, water, and tobacco smoking. Occupational exposure in industries such as battery manufacturing or smelting can lead to significantly elevated body burdens, with regulatory limits established to mitigate toxicity risks	[132–134]

This evaluation requires comparing measured environmental exposure levels with those proven adverse in laboratory studies. Evidence from livestock demonstrates that such overlap is plausible. For instance, concentrations of phthalates such as di(2-ethylhexyl) phthalate (DEHP) detected in commercial pig feed overlap with the ranges used in toxicological studies [135, 136]. In sheep, a low dietary dose of BPA (25 µg/kg/d) led to detectable tissue residues, confirming that environmentally plausible exposures yield measurable internal doses in food-producing animals [137]. Furthermore, the presence of considerable levels of bisphenols and parabens in bovine urine intended for human consumption in India highlights a direct potential for human exposure via the food chain [138]. Biomonitoring extends this evidence to wildlife, showing widespread exposure that correlates with anthropogenic activity. In bottlenose dolphins (*Tursiops truncatus*) from urbanized estuaries, the detection frequency of urinary mono(2-ethylhexyl) phthalate (MEHP) was markedly higher (73.24%) than in those from rural areas (33.33%) [139]. In aquatic systems, while typical riverine BPA concentrations are in the low µg/L range, laboratory studies show that exposure to concentrations as low as 16 µg/L can disrupt fish spermatogenesis, and levels around 100 µg/L induce toxicity in invertebrates such as *Daphnia magna* [140–142]. Similarly, maternal exposure of *Drosophila melanogaster* to environmentally relevant BPA concentrations (0.1–10 mg/L) reduced offspring fecundity transgenerationally [143, 144]. In summary, exposure to environmental estrogens is documented across diverse species. Crucially, environmentally measured concentrations frequently fall within—or closely approach—the dose ranges demonstrated to cause adverse reproductive and developmental effects in controlled laboratory settings. This alignment underscores that experimental hazard data are environmentally relevant and suggests that prevailing exposure levels may pose a tangible risk.

However, inferring risk from this dose alignment must also account for interspecies differences in biological susceptibility. This caution is warranted because the phenotypic conservation described above masks underlying mechanistic complexity: the pathways driving these effects vary substantially across species. Variations in estrogen receptor (ER) subtype distribution, ligand-binding affinity, and downstream signaling pathways critically shape susceptibility and phenotypic outcomes [145–147]. For instance, the distinct repertoire of ER subtypes in teleost fish (ERα, ERβ1, ERβ2) compared to mammals directs tissue-specific responses to EEs [148, 149]. Furthermore, key differences in placental structure, metabolic capacity, and critical windows of developmental sensitivity across species complicate the extrapolation of mechanistic insights from model organisms to wildlife or humans [150–152]. These interspecies nuances underscore that a comparative endocrinology perspective is indispensable for robust hazard assessment and for accurately projecting the ecological and health impacts of EEs. Therefore, acknowledging these mechanistic differences is critical for assessing the weight of evidence: findings from one species cannot be assumed to translate uniformly across taxa, and concordance among multiple species provides the strongest support for causal inference.

Beyond interspecies variability, the transgenerational effects of EEs present another layer of complexity for risk assessment. Through epigenetic mechanisms, EEs may induce effects that persist across generations, altering the expression of reproduction-critical genes by modifying DNA methylation, histone marks, and non-coding RNA regulation, which could lead to adverse outcomes in unexposed offspring. For example, in zebrafish, BPA exposure reduces reproductive output by suppressing the transcription of DNA methyltransferases and promoting genome-wide hypomethylation [153]. While intriguing, these observations must be considered within the context of a still-maturing field. Key questions therefore persist regarding the specificity, stability, and reproducibility of such epigenetic marks across diverse exposures and genetic backgrounds—and, crucially, whether changes characterized in laboratory models ultimately translate to meaningful population-level consequences in wildlife or livestock under complex field conditions.

In summary, the reproductive toxicity of EEs is supported by a multi-layered yet uneven evidence base. The most robust findings emanate from controlled experimental studies, which consistently document EE-induced perturbations in steroidogenesis, gamete quality, and gonadal development across vertebrate species. These experimental data are complemented by epidemiological studies reporting associations between EE exposure and adverse reproductive outcomes in humans. While suggestive, causal interpretation of these associations warrants caution due to inherent methodological limitations. Importantly, human observational data align with mechanistic findings from animal models, together providing stronger support for a causal relationship. In contrast, emerging areas such as transgenerational epigenetic effects remain subject to greater uncertainty, and their full implications require further validation under environmentally relevant conditions. Recognizing this gradation in evidence strength is not merely an academic exercise; it is essential for accurate hazard characterization, prioritizing future research, and designing regulatory strategies that account for both established risks and areas of scientific uncertainty. Thus, a nuanced understanding of this evidence landscape—distinguishing well-established hazards from emerging hypotheses—forms the indispensable foundation for any subsequent effort to evaluate and manage the actual risks these compounds pose.

### Biomarkers of environmental estrogens

Biomarkers serve as critical tools in environmental health research, providing a mechanistic link between external exposure and internal dose, early biological effects, and potential adverse outcomes. For EEs, biomarkers are conventionally categorized into three complementary tiers: biomarkers of exposure, effect, and susceptibility, with the latter often reflected through epigenetic modifications [12, 154]. This hierarchical framework enables a comprehensive assessment, spanning from internal dosimetry to early mechanistic perturbations and long-term regulatory alterations.

Biomarkers of exposure directly quantify the internal dose of a parent compound or its metabolites, typically measured in accessible biological matrices such as urine. Prime examples include bisphenol A glucuronide (BPAG) for BPA exposure and secondary oxidative metabolites (e.g., MEHHP, MEOHP) for phthalates such as DEHP, which are preferred over primary metabolites due to their higher abundance and lower risk of sample contamination [155, 156]. Their validity and high reproducibility are well-established in human biomonitoring studies. However, their short biological half-lives necessitate not only precise analytical methods but also, crucially, carefully timed sample collection for reliable application. Thus, single measurements primarily reflect recent exposure and may not accurately represent cumulative or long-term exposure patterns—a critical consideration for epidemiological study design and interpretation. These methodological considerations directly shape the application of biomonitoring in wildlife and livestock field studies, which simultaneously reveals its utility and inherent challenges. By providing direct evidence of internal dose, biomonitoring effectively links environmental contamination to biological systems. For instance, the detection of phthalate metabolites in bottlenose dolphins not only confirms exposure but also, through metrics such as urinary concentration, facilitates comparison of exposure intensity across populations from contrasting habitats [139, 157]. Similarly, translating quantified DEHP in pig feed into estimated daily intake demonstrates how biomarker-based assessment bridges environmental data with toxicologically relevant doses for risk evaluation [158]. These examples underscore, however, the constraint noted above: the short half-lives of many such biomarkers. While exquisitely sensitive for capturing recent exposure, single measurements are of limited utility for characterizing the chronic, cumulative exposure most relevant to adverse reproductive and developmental outcomes. Therefore, while indispensable for confirming exposure, biomonitoring data must be interpreted with careful consideration of the temporal dynamics linking exposure events to windows of health effect susceptibility.

In contrast, biomarkers of effect indicate measurable early biological alterations triggered by exposure. In aquatic ecotoxicology, the induction of vitellogenin (VTG) in male fish is a highly specific and sensitive biomarker for estrogenic activity, as robustly demonstrated in both laboratory and field studies [159]. VTG's validation has led to its incorporation into standardized regulatory guidelines for endocrine disruptors. At the molecular level, disruption of steroidogenic pathways (e.g., altered aromatase activity) and induction of oxidative stress serve as pivotal, albeit less specific, effect biomarkers that mechanistically link estrogenic exposure to reproductive dysfunction and cellular damage [160, 161]. In contrast to the specificity of VTG, these molecular markers are far less selective; oxidative stress, for instance, represents a generalized response to a wide array of stressors, both estrogenic and non-estrogenic. Consequently, their value is maximized when integrated into a multi-biomarker panel rather than used as standalone indicators.

Building upon the often transient functional alterations captured by effect biomarkers, the third tier of the framework targets more fundamental and potentially persistent molecular mechanisms. Emerging evidence highlights the significance of epigenetic biomarkers, which capture persistent, heritable changes in gene expression potential without altering the DNA sequence itself [162]. These modifications are hypothesized to underpin long-term and transgenerational health risks. Exposure to EEs such as BPA has been associated with global DNA hypomethylation patterns in model organisms [163, 164]. Furthermore, specific alterations in microRNA (miRNA) expression profiles (e.g., miR-146a, miR-27b-3p) have been identified as sensitive indicators of epigenetic disruption, modulating downstream pathways involved in cell cycle control, metabolism, and stress responses [165–167]. Nevertheless, it must be acknowledged that the field of epigenetic biomarkers is still nascent and rapidly evolving. Unlike well-established biomarkers such as VTG, the reproducibility of epigenetic marks across laboratories, exposure regimens, and genetic backgrounds remains an ongoing challenge. Key questions persist regarding both the specificity of these changes for estrogenic exposure versus other stressors and their long-term stability over time and across generations. Therefore, while holding significant promise, epigenetic biomarkers are not yet mature for standalone use in regulatory settings and require rigorous validation across diverse populations and exposure contexts before they can be reliably deployed in such settings.

In summary, the multi-tiered biomarker framework—encompassing exposure, effect, and epigenetic biomarkers—provides a powerful, integrated approach for monitoring and assessing the risks of environmental estrogens. A key consideration is that these tiers differ markedly in their level of validation and practical applicability. While exposure biomarkers and certain highly specific effect biomarkers (e.g., VTG) are well-validated and routinely applicable, other molecular effect markers and, notably, epigenetic biomarkers remain at earlier stages of development, requiring contextual interpretation and further validation, respectively. Recognizing this gradient is important for informing robust study design, guiding accurate data interpretation, and supporting the translational advancement of biomarker science toward regulatory application.

## Mechanisms of endocrine disruption

### Estrogen receptor-mediated signaling mechanisms

**Fig. 2:**
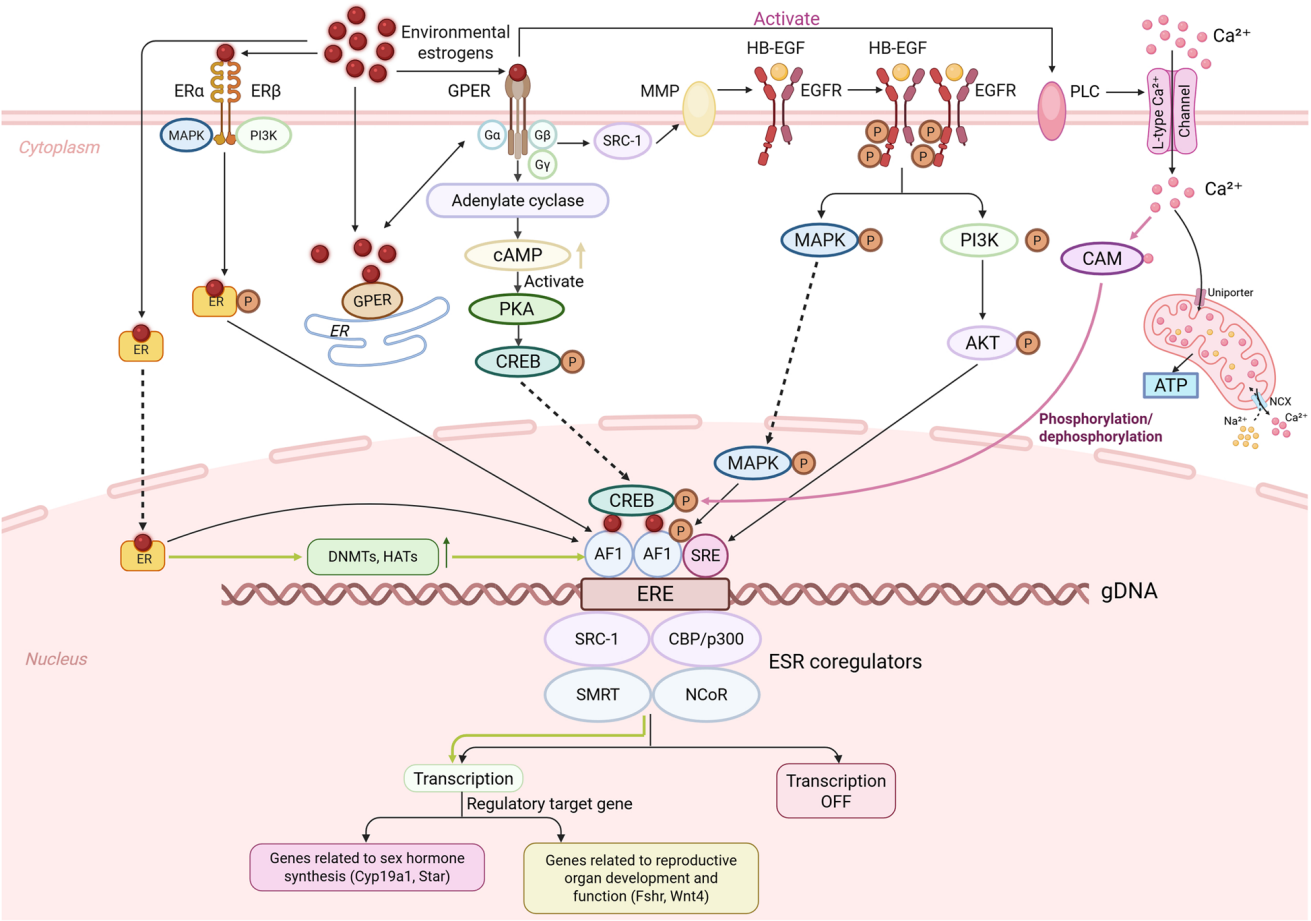
Genomic and non-genomic signaling mechanisms of environmental estrogens (EEs). Environmental estrogens exert their effects primarily through two interconnected signaling modalities mediated by estrogen receptors (ERs). The genomic (nuclear) pathway (left): EEs bind to nuclear estrogen receptors (ERα/ERβ), leading to receptor dimerization, nuclear translocation, and binding to estrogen response elements (EREs) in target gene promoters. This recruits transcriptional co-regulators (e.g., co-activators SRC-1, CBP/p300; co-repressors SMRT, NCoR), regulating the expression of genes critical for reproduction (e.g., *Cyp19a1*, *Star*, *Wnt4*). The non-genomic (rapid) pathway (right): EEs activate the membrane-associated G protein-coupled estrogen receptor (GPER), triggering rapid second messenger cascades. Key downstream axes include the MAPK/ERK and PI3K/Akt pathways, which converge on transcription factors (e.g., CREB) to modulate cell proliferation, differentiation, and survival. Extensive crosstalk exists between these pathways (e.g., GPER-ERα-EGFR axis). Both signaling arms can further induce epigenetic modifications (via DNMTs, HATs/HDACs) and oxidative stress, collectively contributing to the disruptive effects of EEs on physiological systems

The core biological effects of EEs stem from their ability to activate and modulate ERs. ERs are broadly categorized into two classes: nuclear receptors (nER) and membrane receptors (mER). The nERs primarily include ERα (encoded by the *ESR1* gene) and ERβ (encoded by the *ESR2* gene), while the mER is represented by the G protein-coupled estrogen receptor, GPER (also known as GPR30) [168–170]. Owing to their high structural similarity to endogenous estrogens, EEs can competitively bind to ERs, induce conformational changes, and activate downstream signaling pathways, thereby mimicking or amplifying the physiological effects of natural hormones. Recent research acknowledges that ER-mediated effects extend beyond sex hormone regulation to encompass cellular metabolism, immune regulation, and tissue maintenance [169–171]. These genomic and non-genomic signaling pathways are schematically summarized in Fig. 2.

#### Genomic effects mediated by nuclear receptors (nER)

In the canonical genomic pathway, EEs bind to ER subtypes ERα or ERβ, inducing receptor dimerization and translocation to the nucleus, where they recognize estrogen response elements (EREs) in target gene promoters to regulate transcription [169, 170, 172]. This process relies on the recruitment of co-regulators—such as co-activators (SRC-1, CBP/p300) or co-repressors (SMRT, NCoR)—which modulate chromatin accessibility. Core target genes include those involved in steroidogenesis (e.g., *Cyp19a1*, *Star*) and reproductive organ development (e.g., *Hoxa10*, *Wnt4*) [172, 173]. Different EEs exhibit varying binding affinities for ER subtypes. Studies indicate that BPA has a higher affinity for ERβ than ERα, potentially exerting stronger effects in ERβ-rich tissues like the ovary and prostate [168, 170]. Functionally, ERα primarily mediates proliferation signals, whereas ERβ regulates differentiation and inhibits excessive proliferation; their non-redundancy is thought to determine the spatiotemporal heterogeneity of EE toxicity. Beyond direct activation, EEs can amplify disruption through "receptor crosstalk" with Androgen Receptor (AR), Progesterone Receptor (PR), and Thyroid Hormone Receptor (TR), disrupting multiple endocrine axes simultaneously [82, 174, 175]. Furthermore, novel EEs like BPS and BPF can activate ER signaling via allosteric modulation of the N-terminal domain, representing a non-canonical activation mechanism [176]. In summary, EEs exert complex toxic effects through a multi-layered pattern involving receptor binding, genomic signaling activation, and crosstalk with other nuclear receptors.

#### Non-genomic effects mediated by membrane receptors (mER)

Rapid non-genomic effects are mediated by membrane-bound receptors, particularly GPER. Upon activation, GPER initiates second messenger systems such as cAMP/PKA, PLC/Ca^2^^+^, MAPK/ERK, and PI3K/Akt pathways, regulating proliferation, differentiation, and apoptosis [171, 175]. Studies have demonstrated that BPA specifically activates GPER, inducing ERK1/2 phosphorylation in ovarian granulosa cells and upregulating *Cyp19a1*, leading to follicular dysfunction [171]. In males, GPER has been shown to play a role in sperm motility and the acrosome reaction. A bidirectional regulatory mechanism exists between GPER and nuclear ERα: GPER enhances ERα transcriptional activity via the MAPK/ERK pathway and synergistically activates EGFR signaling, establishing an integrated "membrane-to-nucleus" coordination mechanism [171, 175]. Additionally, GPER functions extend to the nervous, cardiovascular, and immune systems, indicating that its aberrant activation by EEs may contribute to multi-systemic disturbances [171, 177].

### Mechanisms of multi-endocrine axis dysfunction

The disruption of multiple endocrine feedback pathways by EEs—encompassing the hypothalamic-pituitary-gonadal (HPG), hypothalamic-pituitary-thyroid (HPT), and hypothalamic-pituitary-adrenal (HPA) axes—represents a pivotal mechanism of systemic reproductive toxicity [178–180]. The HPG axis serves as the primary target, where in EEs can compromise function by altering hypothalamic GnRH neuron excitability, inhibiting Kisspeptin signaling, and suppressing pituitary gonadotropin secretion. Specifically, BPA exposure during puberty has been shown to disrupt sex hormone feedback loops, leading to blunted LH surges and ovulatory dysfunction in females [181, 182]; in males, EEs are associated with impaired Sertoli cell function and attenuate androgen-mediated negative feedback [146–148].

Importantly, this reproductive toxicity may be amplified by the coordinated disruption of thyroid, adrenal, and metabolic axes. Compounds such as BPA interfere with thyroid hormone homeostasis by perturbing receptor signaling and transport protein binding, whereas phthalates disrupt adrenal corticosteroidogenesis [183, 184]. These perturbations collectively remodel the systemic endocrine milieu essential for normal HPG function. Notably, EE-induced metabolic dysfunction—particularly BPA-associated insulin resistance and hyperinsulinemia—is recognized as a key factor contributing to reproductive disorders such as polycystic ovary syndrome (PCOS) [185, 186]. Within this synergistic framework, thyroid and adrenal disturbances alter the permissive hormonal background, while metabolic axis dysfunction provides a direct pathological impetus. This multi-system, cross-axis synergy thus highlights the complex nature of EE-induced reproductive toxicity.

## Epigenetic mechanisms

**Fig. 3:**
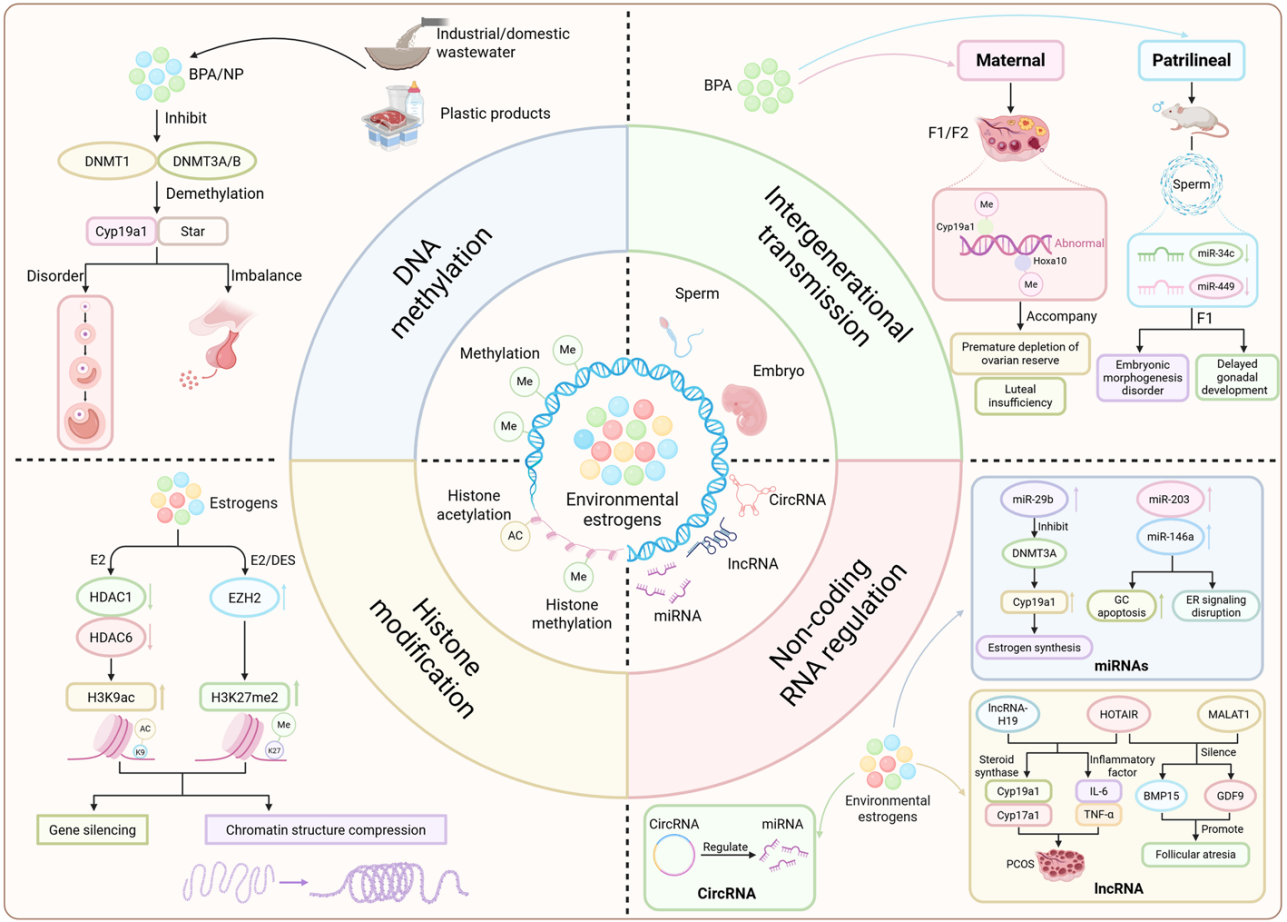
Mechanisms underlying the reproductive and transgenerational toxicity of environmental estrogens (EEs). Environmental estrogens induce reproductive dysfunction and heritable effects by disrupting three core epigenetic pillars. 1) DNA methylation dysregulation (Left): EEs (e.g., BPA) inhibit DNA methyltransferases (DNMT1, DNMT3A/3B), leading to promoter hypomethylation and consequent overexpression of key steroidogenic genes (e.g., *Cyp19a1*, *Star*), thereby disrupting hormonal homeostasis. 2) Histone modification alterations (Middle): EEs modulate the activity of histone-modifying enzymes, such as downregulating HDACs and upregulating the methyltransferase EZH2. This shifts the histone mark balance (e.g., increasing H3K9ac and H3K27me2/3), resulting in chromatin remodeling and aberrant gene silencing that affects gametogenesis and ovarian function. 3) Non-coding RNA network disruption (Right): EEs dysregulate expression of specific microRNAs (e.g., miR-29b, miR-34c, miR-146a) and long non-coding RNAs (e.g., HOTAIR). These ncRNAs post-transcriptionally silence target genes or recruit repressive complexes (e.g., PRC2) to genomic loci, forming regulatory axes (e.g., miR-29b/DNMT3A/Cyp19a1; HOTAIR/PRC2/BMP15) that impair cell cycle control, apoptosis, and steroidogenesis. Collectively, these epigenetic perturbations converge to disrupt gonadal function. Crucially, such acquired alterations in DNA methylation and sperm-borne small RNAs (e.g., miR-34c) can escape germline reprogramming, enabling transgenerational inheritance of reproductive defects (e.g., premature ovarian insufficiency, embryonic failure) through maternal and paternal lineages to F1-F2 offspring

Beyond receptor-mediated effects, EEs also disrupt reproductive health through epigenetic mechanisms, which can confer stability and heritability to toxic effects [186–189]. EEs can modulate the transcriptional activity of reproduction-related genes (e.g., *Esr1*, *Cyp19a1*, *Fshr*) by interfering with DNA methylation, histone modifications, and non-coding RNA expression. These core epigenetic mechanisms and their transgenerational potential are schematically summarized in Fig. 3.

DNA methylation, primarily mediated by DNA methyltransferases (DNMTs), involves the modification of CpG islands in promoter regions. EEs, such as BPA, can inhibit DNMT activity, leading to promoter hypomethylation of genes such as *Cyp19a1* and *Star*, which in turn may trigger abnormal expression and hormone synthesis imbalances [186–188]. In the context of histone modifications, EEs may reshape the epigenetic landscape by modulating enzymes such as histone acetyltransferases (HATs), histone deacetylases (HDACs), and histone methyltransferases (HMTs). For instance, estrogen exposure can downregulate HDACs and upregulate the methyltransferase EZH2, thereby altering chromatin compaction and gene silencing [187]. These modifications have been linked to sperm nuclear protein replacement and follicular activation thresholds, suggesting potential molecular mechanisms underlying phenotypes such as premature ovarian insufficiency (POI) [189].

Notably, epigenetic marks established in germ cells may escape embryonic reprogramming and be transmitted across generations via DNA methylation patterns and non-coding RNAs [186, 189]. Research demonstrates that maternal BPA exposure induces persistent methylation abnormalities in ovarian genes (e.g., *Cyp19a1*, *Hoxa10*) across multiple generations, accompanied by follicular depletion [186]. Paternal exposure also has the potential to establish heritable "epigenetic memory" through sperm-borne small RNAs; specifically, BPA exposure significantly downregulates miR-34c and miR-449 in sperm—microRNAs critical for zygotic genome activation—leading to abnormal embryogenesis and delayed gonadal development in offspring [189, 190].

Furthermore, non-coding RNA networks—including microRNAs (miRNAs), long non-coding RNAs (lncRNAs), and circular RNAs (circRNAs)—play pivotal roles in mediating EE toxicity by regulating gene transcription and chromatin states [187, 189, 191]. Exposure to EEs such as BPA has been linked to upregulated miR-29b, which targets DNMT3A, leading to demethylation of the *Cyp19a1* promoter; similarly, aberrant elevation of miR-203 and miR-146a is associated with granulosa cell apoptosis [186, 188]. Regarding lncRNAs, dysregulation of molecules such as HOTAIR correlates with inflammatory cytokine levels and steroidogenic enzyme disruption in patients with polycystic ovary syndrome (PCOS) [191, 192]. Mechanistically, these lncRNAs can recruit repressive complexes such as polycomb repressive complex 2 (PRC2) to the promoters of key genes (e.g., *BMP15*), inducing chromatin condensation and follicular atresia [189].

## Oxidative stress and inflammatory responses

**Fig. 4:**
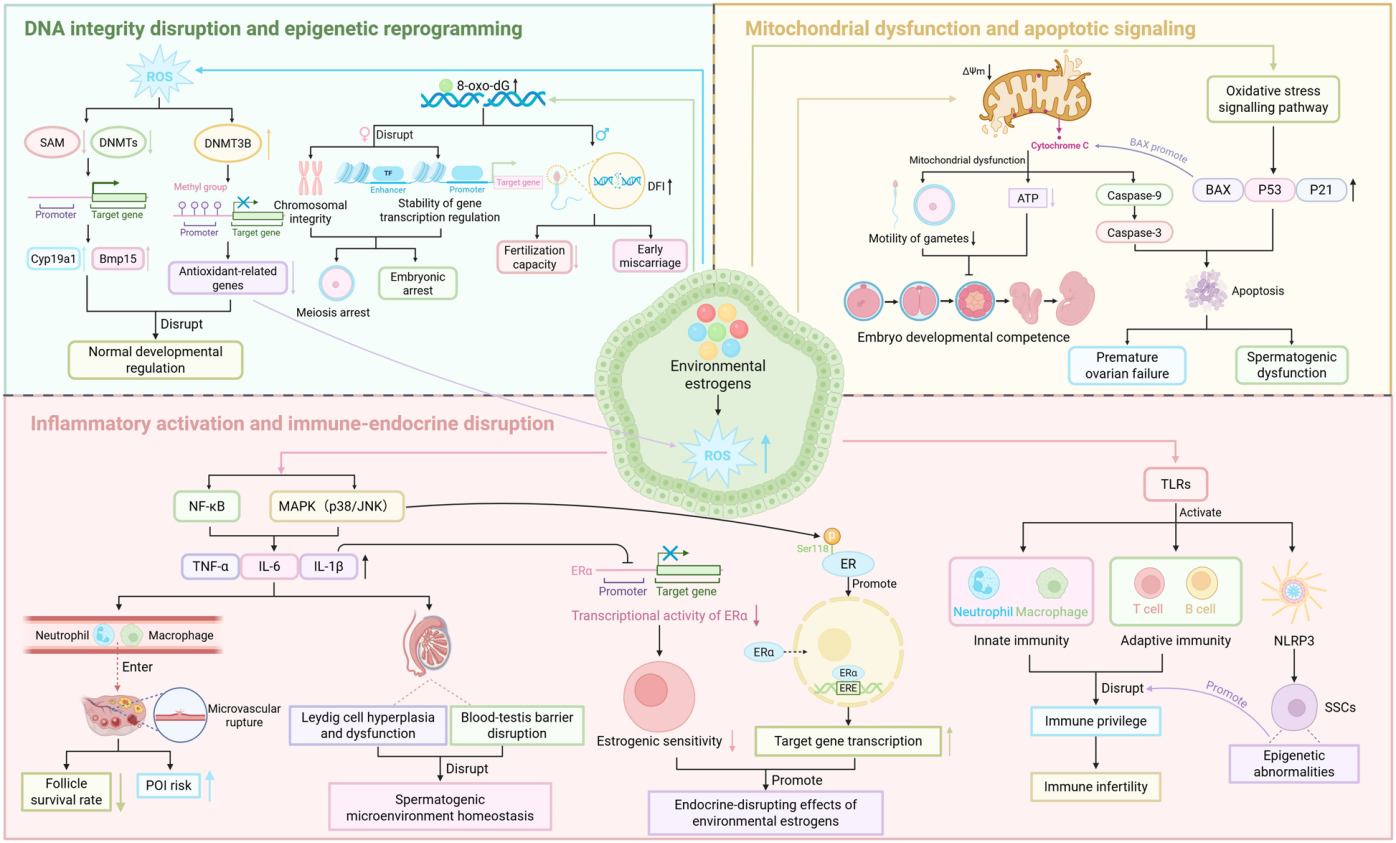
ROS-centered mechanistic model of reproductive toxicity induced by environmental estrogens(EEs). Environmental estrogens initiate a self-perpetuating cycle of "oxidative damage and inflammatory response" that compromises reproductive health. This central process drives impairment through three interconnected pathways: (1) Genomic and Epigenetic Instability: ROS directly causes DNA damage and disrupts epigenetic regulation (e.g., via DNMTs), leading to aberrant gene expression and impaired embryonic development. (2) Mitochondrial Apoptosis: ROS induces oxidative stress and mitochondrial dysfunction, activating the caspase cascade (e.g., Caspase-9, Caspase-3) and promoting germ cell apoptosis. (3) Microenvironment Disruption: ROS activates NF-κB and MAPK signaling, upregulating pro-inflammatory cytokines (e.g., TNF-α, IL-6, IL-1β) and disrupting critical reproductive structures like the blood-testis barrier and immune privilege. The convergence of these pathways culminates in adverse outcomes including premature ovarian insufficiency, spermatogenic defects, and immune infertility, underscoring the multifaceted toxicity of EEs

Oxidative stress and inflammatory responses are recognized as core molecular mechanisms underlying EE-induced reproductive toxicity. Environmental estrogens elevate intracellular reactive oxygen species (ROS) levels, disrupting redox homeostasis and potentially establishing a vicious "oxidative damage–inflammatory response" cycle [187, 193, 194]. This proposed ROS‑centered mechanism is schematically summarized in Fig. 4.

At the cellular level, germ cells exhibit high susceptibility to oxidative stress. Low-dose EE exposure can rapidly induce mitochondrial membrane potential collapse and caspase cascade activation, initiating apoptosis [195–197]. Specifically, BPA increases intracellular ROS, causing mitochondrial structural damage and accelerating apoptotic signaling; concurrently, ROS-induced adducts, such as 8-oxo-dG, compromise chromosomal integrity, increasing the risks of meiotic arrest and sperm DNA fragmentation [187, 198, 199]. Furthermore, mitochondrial dysfunction suppresses ATP synthesis, compromising gamete motility. Beyond direct cellular damage, ROS can modulate DNA methylation by depleting the methyl donor S-adenosylmethionine (SAM), thereby providing a mechanistic link between oxidative stress and epigenetic dysregulation [187, 200–202].

This pro-oxidant microenvironment subsequently activates multiple inflammatory signaling pathways, including NF-κB and MAPK (p38, JNK). Specifically, BPA has been shown to activate NF-κB via ROS accumulation, leading to leukocyte infiltration and vascular damage in ovarian tissue, while in males, this mechanism contributes to Leydig cell dysfunction and blood-testis barrier disruption [193, 198, 203]. The resulting upregulation of pro-inflammatory cytokines (IL-6, IL-1β, TNF-α) not only establishes a chronic inflammatory microenvironment but also enhances TLR-mediated signal transduction, disrupting immune tolerance barriers [204]. Recent studies further indicate that inflammation can activate the NLRP3 inflammasome, inducing epigenetic modifications in spermatogonial stem cells and thereby potentially amplifying toxic risk across generations [205–207].

## Detoxification and mitigation strategies

Environmental estrogens disrupt reproductive system development and function through multiple targets and mechanisms, producing toxic effects characterized by long latency periods, low-dose sensitivity, and transgenerational inheritance. Consequently, identifying exposure-related biomarkers, elucidating key effector pathways, and developing targeted interventions have become central priorities in reproductive toxicity risk assessment and safety prevention. Recent research has evolved from initial exposure identification and sensitive target screening to systematic investigation of molecular intervention mechanisms, progressively establishing an integrated prevention framework that equally emphasizes biomonitoring and functional protection. This provides both theoretical foundation and practical support for effectively mitigating EE-induced reproductive toxicity.

As the understanding of EE mechanisms deepens, translating this knowledge into operational strategies for risk assessment necessitates a framework that systematically links exposure to adverse outcomes. This translation is best operationalized by constructing a multi-tiered, biologically anchored biomarker system designed to capture key events along the toxicity pathway [208]. Such a system advances beyond merely quantifying internal dose by integrating key mechanistic nodes. For instance, dysregulation of gonadal estrogen receptor (ERα/ERβ) expression and function constitutes a pivotal molecular initiating event, which directly informs the development of mechanistically grounded in vitro screening models [209, 210]. Concurrently, early adverse phenotypic outcomes—such as reduced follicular reserve or decreased sperm concentration—serve as biologically anchored endpoints that link the assessment to functional health consequences [211–213]. By linking molecular perturbations to apical outcomes, this integrated approach is fundamental for advancing from exposure identification toward mechanism-based risk assessment and early warning, thereby establishing the diagnostic framework upon which targeted interventions can be rationally designed.

Building directly on this framework, targeted interventions can be developed to address the core toxicological mechanisms thus identified—oxidative stress, hormonal disruption, inflammatory responses, and epigenetic abnormalities. Among these, antioxidant-based strategies represent the most substantiated approach. Classic reactive oxygen scavengers—such as vitamin E, glutathione, and N-acetylcysteine (NAC)—have been extensively validated across animal and cellular models, demonstrating reproducible efficacy in reducing oxidative stress, restoring mitochondrial function, and mitigating toxic phenotypes such as premature ovarian insufficiency and spermatogenic defects [214, 215]. Despite their well-documented efficacy in controlled experimental settings, the translational outcomes of antioxidant interventions remain inconsistent and context-dependent. Notably, while vitamin E supplementation confers protection against BPA-induced testicular damage in healthy rodents, this benefit does not uniformly extend to other toxicants or metabolic conditions. Specifically, recent evidence indicates that vitamin E fails to ameliorate BPS-induced testicular injury in diabetic models and may even potentiate toxicity, challenging the assumption of universal cytoprotection [216, 217]. Furthermore, high-dose antioxidant supplementation raises significant safety concerns; excessive intake of specific agents, such as vitamin E, has been linked to adverse outcomes in human clinical trials, including an elevated risk of hemorrhagic stroke and all-cause mortality [218, 219]. Collectively, these observations underscore the critical need to establish safe and effective dose windows, suggesting that the therapeutic window for antioxidant interventions may be narrower than initially anticipated.

In contrast, evidence for other promising interventions remains more preliminary. Certain natural polyphenols (e.g., resveratrol, quercetin, rutin) exert effects beyond antioxidant activity, including the modulation of estrogen receptor expression and influence on epigenetic regulators such as HDACs and DNMTs, thereby potentially improving oocyte quality and embryonic development. Similarly, direct targeting of epigenetic pathways with HDAC inhibitors or DNMT modulators has shown promise in animal models for reversing gene expression alterations and promoting ovarian function recovery [220, 221]. However, the clinical translation of epigenetic modulators faces substantial hurdles. Many HDAC inhibitors and DNMT modulators currently approved for oncology indications exhibit significant off-target effects and systemic toxicity, precluding their long-term administration for reproductive health indications in otherwise healthy populations [222, 223]. Furthermore, the developmental stage-specificity of epigenetic programming raises concerns that interventions applied during inappropriate windows may inadvertently disrupt, rather than restore, normal reproductive function [224]. Collectively, these safety and specificity challenges necessitate the development of targeted delivery systems and the identification of more selective epigenetic modifiers before such approaches can be considered for routine application. These concerns are particularly pertinent for pleiotropic compounds like resveratrol, whose biological effects extend well beyond simple antioxidant activity, further complicating the prediction of their therapeutic index.

To bridge the gap between mechanistic insights and practical application, a spectrum of complementary strategies—ranging from immediate nutritional tools to longer-term biotechnological solutions—warrants consideration. Nutritional supplementation represents a viable and readily deployable approach. Beyond classic antioxidants, micronutrients such as zinc have demonstrated protective efficacy; for instance, zinc supplementation alleviates BPA-induced reproductive toxicity in mice by inhibiting ferroptosis and apoptosis while restoring zinc homeostasis [225]. In production systems such as aquaculture and livestock farming, incorporating functional additives—including antioxidant compounds (e.g., selenium yeast, vitamins C and E) or specific probiotics—has proven effective in mitigating gonadal abnormalities induced by environmental estrogens and supporting reproductive performance under stress [226, 227]. Refined management practices are fundamental for primary prevention. Essential measures include replacing plastic water lines with stainless steel or glass alternatives, using low-leaching materials for bedding and storage, and instituting routine monitoring of water and feed to minimize EE input [216, 228]. Biomarker-based assessments have identified animal feed as a significant exposure vector for environmental estrogens; therefore, mitigation strategies should prioritize feed safety [229]. Contamination primarily stems from plastic packaging, processing equipment, and contaminated raw materials along the supply chain [230]. Consequently, comprehensive strategies must extend beyond routine monitoring to include: (i) establishing maximum residue limits (MRLs) for estrogenic chemicals in animal feed; (ii) replacing plastic-containing equipment with stainless steel alternatives throughout production lines; and (iii) sourcing raw materials from suppliers with verified low-contamination profiles. These targeted interventions aim to mitigate a key and manageable exposure pathway at its source, thereby complementing broader environmental remediation efforts. Furthermore, given the association between EE exposure, gut microbiota dysbiosis, and testicular dysfunction, proactive management of intestinal health through dietary prebiotics or probiotics represents a promising supportive strategy for maintaining systemic homeostasis [231, 232].

To achieve long-term resilience, genetic and microbiome-targeted avenues offer significant potential. Future breeding programs could leverage genomic tools, such as genome-wide association studies, to identify traits associated with tolerance to specific EEs, thereby enhancing reproductive robustness in contaminated environments [233]. More directly, gene-editing tools such as CRISPR/Cas9 enable precise modification of key genes within estrogen response pathways [234]. Proof-of-concept studies in model organisms have demonstrated that this approach can reduce susceptibility to EE-induced reproductive impairment, paving the way for developing less susceptible animal lines [235]. Modulating the gut microbiome—a critical hub within the gut-testis axis—introduces another innovative intervention layer. Specific probiotic supplementation has exhibited direct protective efficacy; for instance, in zebrafish, a probiotic consortium (SLAb51) mitigated BPA-induced deficits in oocyte growth, vitellogenin expression, and spermatogenic signaling, ultimately improving reproductive capacity [236]. However, translating these encouraging preclinical findings into practice presents substantial challenges. Probiotic efficacy is highly variable, dependent on strain specificity, dosage regimens, and host factors such as baseline microbiota composition and immune status [237]. Consequently, clinical studies investigating probiotic interventions for reproductive outcomes have yielded inconsistent results, with some trials failing to replicate the protective effects observed in animal models [238]. Beyond biological heterogeneity, the regulatory landscape for probiotic feed additives remains fragmented across jurisdictions, characterized by divergent requirements for safety assessment, efficacy demonstration, and product labeling [239, 240]. These regulatory ambiguities, compounded by the current lack of standardized potency assays, hinder the commercialization and widespread adoption of microbiome-targeted interventions in animal agriculture.

Given these differences in the strength of evidence, a staged approach to implementation is warranted, prioritizing near-term integration of validated tools while investing in next-generation biotechnologies. Ultimately, achieving robust and lasting protection requires a comprehensive, system-level approach to exposure control that extends beyond specific interventions. An effective framework rests on three pillars: source control, environmental remediation, and individualized risk assessment. Source control requires stringent regulation of high-risk chemicals (e.g., BPA, nonylphenol, phthalates) in food packaging, medical devices, and consumer goods [37]. For environmental remediation, a diverse toolkit exists to intercept EEs in the environment. As summarized in Table 3, this includes enzymatic degradation, physical adsorption, advanced oxidation processes, bioremediation, and phytoremediation, each with specific applications for different pollutants and contexts. However, their performance in real-world environments—characterized by complex contaminant mixtures and variable physicochemical conditions—must be validated through field-scale studies. At the individual level, incorporating susceptibility factors (e.g., species, sex, physiological status) could enable precise risk identification and stratified interventions. Nevertheless, translating this conceptual approach into practice depends on the development of validated susceptibility biomarkers and user-friendly risk stratification tools. Collectively, these strategies embody a forward-looking paradigm of "precision identification, systematic management, and tailored intervention", thereby enhancing the systemic safeguarding of reproductive health [250].

**Table 3:** Summary of primary remediation technologies for environmental estrogens and their application conditions

Remediation technology	Specific method/reagent	Target pollutant(s)	Operating conditions/dosage	Reference
Enzymatic degradation	Laccase (Trametes versicolor)	Bisphenol A (BPA)	0.1–0.2 U/mL (pH 5.0, 25 °C, 1 h)	[241]
	Laccase-Mediator System (Laccase + HBT)	Bisphenol A (BPA)	Requires redox mediators (e.g., 1 mmol/L HBT)	[242]
	Specific Hydrolase (ZHD101 lactonohydrolase)	Zearalenone (ZEA)	37 °C, pH 8.0; Enzyme activity: 1 U = degradation of 1 μg ZEA/min at 37 °C	[243]
Physical adsorption	Powdered Activated Carbon (PAC)	Bisphenol A (BPA)	0.3 g/L (typical dose, adjust based on water quality)	[244]
	Biochar (BC)	Cadmium (Cd), Lead (Pb)	Dose depends on biochar type and pollution level (typically 5–10 g/L)	[245]
Advanced oxidation processes	Photo-Fenton (Fe^2+^ + H_2_O_2_ + UV)	Polychlorinated Biphenyls (PCBs)	Fe^2+^ 0.1 mmol/L, H_2_O_2_ 50 mmol/L, UV irradiation	[246]
Bioremediation	Microbial Consortia (*Pseudomonas* spp.)	DDT/DDE	5% inoculum	[247]
	Fungi (White-Rot Fungi)	Polychlorinated Biphenyls (PCBs)	10^6^ spores/mL	[248]
Phytoremediation	Aquatic Plants (Water Hyacinth)	Bisphenol A (BPA)	10–20 plants/m^2^ (or ~5 g dry weight/L, 14–28 d)	[249]

In summary, mitigating EE-mediated reproductive toxicity requires moving from isolated interventions toward an integrated strategy that combines readily deployable tools with innovative biotechnological and microbiome-based solutions. Crucially, the marked heterogeneity in evidence maturity across these intervention tiers dictates a corresponding gradient in recommendation confidence. Acknowledging this gradation in evidence strength is pivotal not only for managing implementation expectations but also for channeling research investment toward bridging the critical knowledge gaps that currently constrain the translational potential of advanced interventions. Future research must prioritize validating the cost-effectiveness and long-term efficacy of these integrated strategies in real-world settings, as well as developing lifecycle-stage-specific intervention guidelines. Ultimately, addressing the multifactorial nature of EE toxicity requires a systematic framework rooted in the concurrent identification of molecular targets, elucidation of pathogenic mechanisms, and coordination of intervention pathways. This approach enables targeted interventions at each stage—from source to exposure to health outcome. A comprehensive risk management roadmap must, therefore, strategically combine receptor-based biological interventions with the source-directed environmental remediation technologies detailed in Table 3, to effectively break the exposure chain at multiple points.

## Conclusion

This review has systematically examined the categories, mechanisms, hazards, biomarkers, and mitigation strategies associated with EEs. The evidence varies considerably across these domains in both strength and maturity, with direct implications for risk assessment and intervention design.

The most robust and consistent evidence derives from controlled experimental studies, which have conclusively demonstrated that EEs can impair reproductive function in diverse vertebrate species. Key established mechanisms include interference with estrogen receptor signaling, induction of epigenetic modifications, and promotion of oxidative stress and inflammatory responses. These mechanistic insights are supported by validated biomarkers (e.g., vitellogenin induction in fish), some of which are now integrated into regulatory screening frameworks. Epidemiological studies have reported associations between EE exposure and adverse reproductive outcomes, such as reduced semen quality and altered pubertal timing. However, inherent methodological limitations—including potential confounding, exposure misclassification, and population heterogeneity—constrain causal inference from human data alone and therefore warrant cautious interpretation of these findings.

Several interconnected knowledge gaps currently hinder the translation of mechanistic data into robust risk assessment paradigms. First, substantial interspecies differences in estrogen receptor biology, metabolic capacity, and critical windows of susceptibility complicate extrapolation from model organisms to humans and wildlife. While phenotypic responses are broadly conserved, the underlying molecular mechanisms can exhibit significant divergence, and quantitative frameworks for cross-species extrapolation remain underdeveloped. Second, emerging areas such as transgenerational epigenetic effects and microbiome-mediated toxicity are supported by compelling but preliminary evidence, primarily from a limited set of model organisms under controlled laboratory conditions. Their relevance under environmentally realistic (e.g., low-dose, mixture) exposure scenarios remains uncertain. Key questions persist regarding the specificity, stability, and reproducibility of epigenetic marks across exposures and genetic backgrounds, and whether laboratory-observed effects translate to population-level consequences in the field. Third, the prevailing focus on single-compound toxicology represents a critical limitation, as real-world exposures invariably involve complex mixtures with potential additive, synergistic, or antagonistic interactions. Consequently, understanding of cumulative health risks from combined EE exposures remains inadequate, undermining the ecological validity of current hazard data. Finally, the translational readiness of proposed mitigation strategies varies markedly. Nutritional and management interventions are supported by sufficient evidence for near-term implementation, whereas genetic, epigenetic, and microbiome-targeted approaches remain largely at the proof-of-concept stage, requiring rigorous field trials to evaluate their efficacy, safety, and cost-effectiveness under real-world conditions.

Addressing these gaps demands a coordinated research agenda. Priorities should include: (i) long-term, multigenerational exposure studies employing environmentally relevant doses and mixtures, integrated with multi-omics technologies; (ii) development of improved quantitative frameworks for cross-species extrapolation, combining comparative endocrinology with toxicokinetic-toxicodynamic modeling; (iii) rigorous validation of emerging biomarkers (e.g., epigenetic, microbiome-based) across diverse populations and exposure contexts to establish their specificity and reproducibility for regulatory use; and (iv) field-scale evaluation of all proposed interventions—from nutritional to biotechnological—with emphasis on dose optimization, long-term safety, scalability, and regulatory harmonization for novel approaches such as probiotic feed additives.

In summary, this synthesis consolidates current understanding of EEs and explicitly demarcates areas of strong evidence from those requiring further investigation. This critical perspective is essential for developing targeted, effective, and scientifically defensible strategies to mitigate the potential risks posed by environmental estrogens to reproductive health.

## Data Availability

No datasets were generated or analysed during the current study.

## Abbreviations

AhR: Aryl hydrocarbon receptor
AR: Androgen receptor
BPA: Bisphenol A
BPAF: Bisphenol AF
BPF: Bisphenol F
BPS: Bisphenol S
Cyp19a1: Cytochrome P450 family 19 subfamily A member 1 (aromatase)
DDE: Dichlorodiphenyldichloroethylene
DDT: Dichlorodiphenyltrichloroethane
DEHP: Di(2-ethylhexyl) phthalate
DNMTs: DNA methyltransferases
E2: 17β-Estradiol
EE2: 17α-Ethinylestradiol
EEs: Environmental estrogens
ERα: Estrogen receptor alpha
ERβ: Estrogen receptor beta
EREs: Estrogen response elements
GPER: G protein-coupled estrogen receptor
HPG: Hypothalamic-pituitary-gonadal
lncRNAs: Long non-coding RNAs
MAPK: Mitogen-activated protein kinase
mER: Membrane estrogen receptor
miRNAs: MicroRNAs
nER: Nuclear estrogen receptor
PCBs: Polychlorinated biphenyls
PCOS: Polycystic ovary syndrome
PI3K: Phosphatidylinositol 3-kinase
PPARγ: Peroxisome proliferator-activated receptor gamma
PR: Progesterone receptor
ROS: Reactive oxygen species
TR: Thyroid hormone receptor
VTG: Vitellogenin
ZEA: Zearalenone
3β-HSD: 3β-Hydroxysteroid dehydrogenase
17β-HSD: 17β-Hydroxysteroid dehydrogenase
